# Beliefs and desires in the predictive brain

**DOI:** 10.1038/s41467-020-18332-9

**Published:** 2020-09-02

**Authors:** Daniel Yon, Cecilia Heyes, Clare Press

**Affiliations:** 1 Department of Psychology, Goldsmiths, University of London, SE14 6NW London, UK; 2 Department of Psychological Sciences, Birkbeck, University of London, WC1E 7HX London, UK; 3 All Souls College, University of Oxford, OX1 4AL Oxford, UK

**Keywords:** Cognitive neuroscience, Computational neuroscience, Psychology

## Abstract

Bayesian brain theories suggest that perception, action and cognition arise as animals minimise the mismatch between their expectations and reality. This principle could unify cognitive science with the broader natural sciences, but leave key elements of cognition and behaviour unexplained.

In everyday life, we tend to explain the behaviour of ourselves and other creatures in terms of beliefs and desires. For example, we might say that a rat pulls a lever or a scientist runs an experiment because they believe that certain outcomes will ensue (e.g. a piece of food or a piece of data) and because these are outcomes they desire (e.g. because they are hungry or curious).

The idea that action is motivated by belief-like and desire-like representations—respectively defining which states of the world are most probable and most valuable (Box 1)—is also a key feature of theories across the cognitive sciences. For example, cognitive models suggest goal-directed action depends on separate associations between actions and outcomes (instrumental beliefs) and outcomes and values (incentives)^1,2^. A similar distinction is fundamental to models of economic choice, where decisions are thought to reflect a combination of utilities (how good is this option?) and probabilities (how certain am I to obtain it?)^3^.

## Box 1

Belief-like and desire-like mental states

What are belief-like and desire-like states? In colloquial use beliefs and desires are typically thought of as explicit personal-level propositional representations—sentences in the head, such as “I think my keys are in my pocket”, “I want to go to bed”. However, in this article and in line with many cognitive scientists, we assume that belief-like and desire-like states can be subpersonal and/or implicit (e.g. patterns of activity in sensory systems, loss functions in reward systems).

We contend that explaining behaviour requires a clear distinction between belief-like states and desire-like states, where the former track what is probable about the world and the latter track what is valuable to the agent. Mental states differ in their direction-of-fit^17^—whereas belief-like states have a mind-to-world direction (i.e. it is adaptive for agents when their beliefs are adjusted to fit the world), desire-like states have a world-to-mind direction (i.e. it is adaptive for agents when the world matches their desires). In the desert landscape envisaged by the above predictive processing accounts, this distinction is dissolved and behaviour is explained in terms of a single predictive model that can both adjust the world and be adjusted by it. Critics have argued that predictions seem to lack the motivational force needed to work as desires without implausible assumptions about the number or specificity of predictions wired into the model^18^, though proponents of these predictive processing accounts argue this issue can be solved by flexibly adjusting the weight on information flowing in different directions throughout the cortical hierarchy (but see Box 2).

**Fig. 1:**
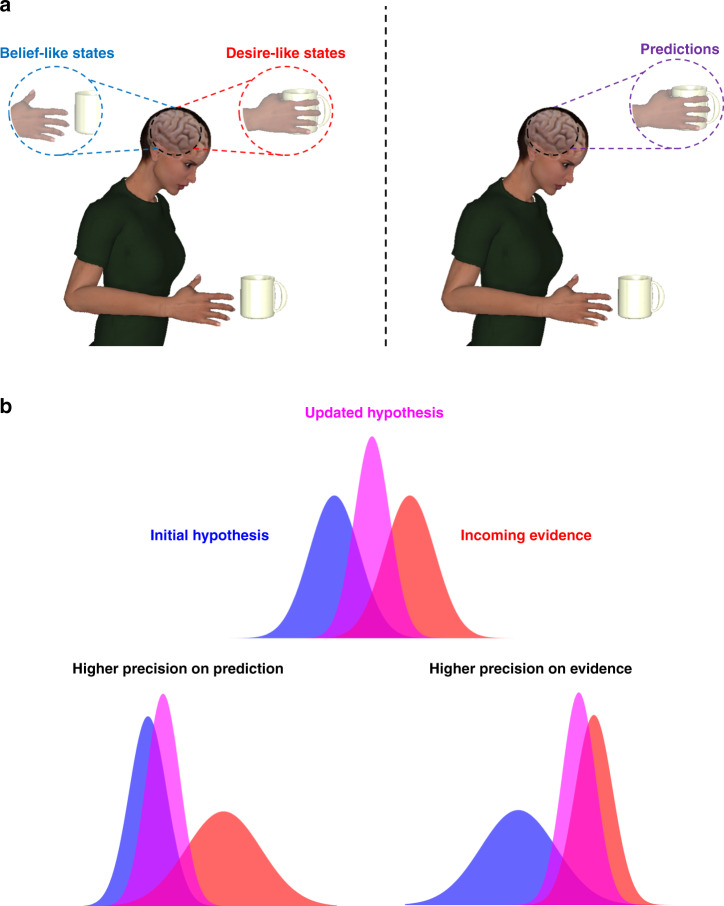
Beliefs, desires, predictions and precision. **a** Left: Classic approaches across the cognitive sciences assume that behaviour is controlled by separate mechanisms representing likely (belief-like) and valuable states of the world (desire-like). Right: However, recent predictive processing models assume behaviour can be explained entirely in terms of predictions—describing a desert landscape view of the mind that dispenses with goals, drives and reward. **b** Predictive processing accounts suggest we refine our internal models of the world by combining initial hypotheses with incoming evidence. In these theories, how (or whether) our hypotheses become updated depends on beliefs about the precision of these two quantities. When agents believe prior predictions are more precise than incoming evidence (bottom left) hypotheses are stubborn and more closely resemble our initial expectations^19^. Conversely, when agents believe sampled evidence is more precise (bottom right), incoming signals have a larger impact on subsequent hypotheses about the world (Box 2).

However, recently cognitive scientists have explored the possibility that the familiar double act of beliefs and desires can be replaced by theories that explain behaviour using only one kind of internal state: prediction (Fig. 1)^4^. These predictive processing accounts based on the free energy principle^5^ assume that the brain acts as a model of the extracranial world, optimised to fit information arriving at the senses. According to this view, the brain is structured in a hierarchical way such that higher cortical areas embody hypotheses about the activity expected in lower areas, which in turn send information up the processing hierarchy signalling the mismatch or ‘error’ between prediction and reality. This structure allows the brain to optimise its fit to the outside world through two kinds of process or ‘inference’. The first is perceptual inference, where incoming sensory signals are used to adjust hypotheses at higher levels, such that the hypotheses more closely match the outside world. The second is active inference, where strong top-down predictions engage muscles and organs to drive action, changing states of the body and the world such that they conform with the prior predictions. More simply put, the brain can either revise its predictions to match the world or change the world to make the predictions come true.

Proponents of this view^4^ suggest that these models leave us with a desert landscape view of cognition, where mental states once thought to be crucial in explaining behaviour—such as goals, drives and desires—are reduced to predictions. Under this account “there is no essential difference between goals or desires and beliefs or predictions”^6^ and “desired outcomes [are] simply…those that an agent believes, a priori, it will obtain”^7^. According to this view, the hungry rat presses the lever because it expects itself to press, since it expects not to be hungry in the future. Neuroscientists and philosophers defending these models have recently reaffirmed that desires emerge as webs of prior beliefs^8^, dissolving the distinction between beliefs and desires: “from motor control to expected utility theory…as each of these constructs is absorbed…the landscape of explanations becomes progressively deserted. Is this something to be celebrated or resisted^9^?”

The predictive processing scheme has the potential to unify cognitive science with other life and social sciences through a common set of principles. For example, it can be shown that any plausible biological system—whether brain, bacterium or birch tree—behaves as though it possesses a predictive model of its environment, and acts in ways that improve the fit between this model and the outside world^10^. It has also been suggested that the same mathematical principles can explain cultural evolution^11^. These models are useful to scientists who seek continuity between the principles explaining human and animal behaviour and those explaining the rest of the natural world.

However, the unifying potential of such predictive processing models may come at a cost to explanatory power. There may still be good reasons for the cognitive scientist to retain the concepts of belief-like and desire-like states in their theoretical arsenal. For example, predictive processing models of active inference assume that we act by generating (false) predictions about the states of our body (e.g. my hand is over there) and enslaving peripheral reflexes to make the prediction come true (i.e. move it). While this formulation provides an elegant account of how motor commands are generated and unpacked in the spinal cord, and there would be little dispute that goals are achieved through error-minimisation processes^12^, a key component of this scheme is the assumption that agents suspend perception of their actions until their predictions are realised—reducing the weight or ‘precision’ afforded to incoming sensory signals^13^ (Box 2). This assumption is required because one state plays the role of belief and desire—I cannot simultaneously represent with one state that my hand is by my side, and that I would like it to be grasping the mug. These assumptions are difficult to reconcile with evidence that agents can simultaneously act and perceptually monitor their actions as they unfold, for example, when adapting to unexpected perturbations in a visually guided reaching movement^12^. It is unclear if there is a straightforward solution to this problem. This kind of sensory-guided goal-directed action is compatible with there being some levels in the hierarchy that do not distinguish between belief-like and desire-like information^1,11^ but not with the absence of this distinction at all levels.

## Box 2

Precision-weighting predictions and evidence

Predictive processing models have often likened the brain to a scientist, suggesting that it generates hypotheses about the outside world (in the form of predictions) which are tested against data (sensory evidence)^19^. Recent predictive processing models deploy the idea of precision-weighting, such that the weight or precision afforded to top-down predictions or bottom-up evidence can be flexibly adjusted. For example, when precision on sensory evidence is high, incoming signals are given more weight in updating hypotheses. This may be adaptive if incoming evidence is especially strong, if agents find themselves in new environments without strong expectations, or if they suspect the world and its contents might rapidly change. In contrast, when precision on the predictions is high, hypotheses are stubborn and insensitive to incoming data. This may be adaptive when incoming sensory evidence is noisy or ambiguous, agents are very confident about what to expect, or they believe the world is likely to be stable—such that predictions based on the past will apply in the future.

Predictive processing models depend on the idea of precision-weighting to explain the action, assuming that agents have predictions (e.g. “I am holding the cup”, “I do not have low blood sugar”) that are assigned especially high precision^6^. Assigning an especially strong weight to such a prediction means that the mismatch between expectation and reality (the prediction error) is resolved by engendering actions (e.g. grasping, eating) rather than changing the prediction. However, affording these predictions high precision necessarily means reducing the precision afforded to evidence that could update them. In other words, precision in these models is zero-sum^20^: assigning more weight to a top-down prediction is equivalent to assigning less weight to bottom-up evidence (and vice versa)^19^. As such, predictions that operate as desires cannot simultaneously operate as (evidence-sensitive) beliefs.

Retaining the distinction between belief-like and desire-like states may also help clinical scientists explain atypical aspects of action. For example, studies of drug addiction have shown that individuals can expect substances to be unrewarding, yet still feel strong compulsions to consume them, with expectations about the pleasantness of consumption (‘liking’) and about one’s future actions (‘wanting’) subserved by dissociable mechanisms^14^. A similar distinction may be important in obsessive-compulsive disorder, where individuals feel strong urges to perform actions they believe to be causally impotent^15^. Such experiences are difficult to explain without distinguishing desire-like and belief-like mechanisms (Box 1).

The predictive processing framework is used by many scientists, and it may be that some are implicitly committed to the belief-desire distinction despite the ‘desert landscape’ view emphatically defended by some of the framework’s key architects^6^. We propose it is important to retain a clear distinction between beliefs and desires when explaining cognition and behaviour. Intriguingly, this distinction could be explicitly reintroduced into predictive processing via the concept of deep temporal models^16^. These accounts propose that agents can act in ways that minimise future prediction errors, possessing separate predictions about states of the world and predictions about plausible actions they could perform. However, while it may be tempting to identify the former and latter types of predictions as beliefs and desires, theorists have not explicitly or implicitly taken steps in this direction. We would welcome such steps, but they would imply that the aim of unifying scientific explanation via the concept of error-minimisation can be only partially achieved. The desert landscape of cognition is not as featureless as it seems, and we must accept that there is a discontinuity between different types of mental state, and between error-minimising systems that possess predictions about the future (e.g. animals) and those that do not (e.g. viruses).

In conclusion, prominent predictive processing models have suggested it is possible to abandon traditional concepts of belief and desire, explaining all cognition and behaviour in terms of predictions. This account holds promise for uniting the study of the mind with the study of the natural world, but discarding these concepts may limit cognitive science’s ability to explain the subtleties of motivated action in health and disease. Though both beliefs and desires could be crafted from the sands of a desert landscape, the cognitive scientist may still find them to be as different as concrete and glass.
